# 

**DOI:** 10.1192/bjb.2021.14

**Published:** 2021-08

**Authors:** Peter Tyrer

**Affiliations:** Emeritus Professor of Community Psychiatry in the Division of Psychiatry, Imperial College, Hammersmith Hospital, London, UK. Email: p.tyrer@imperial.ac.uk

**Figure:**
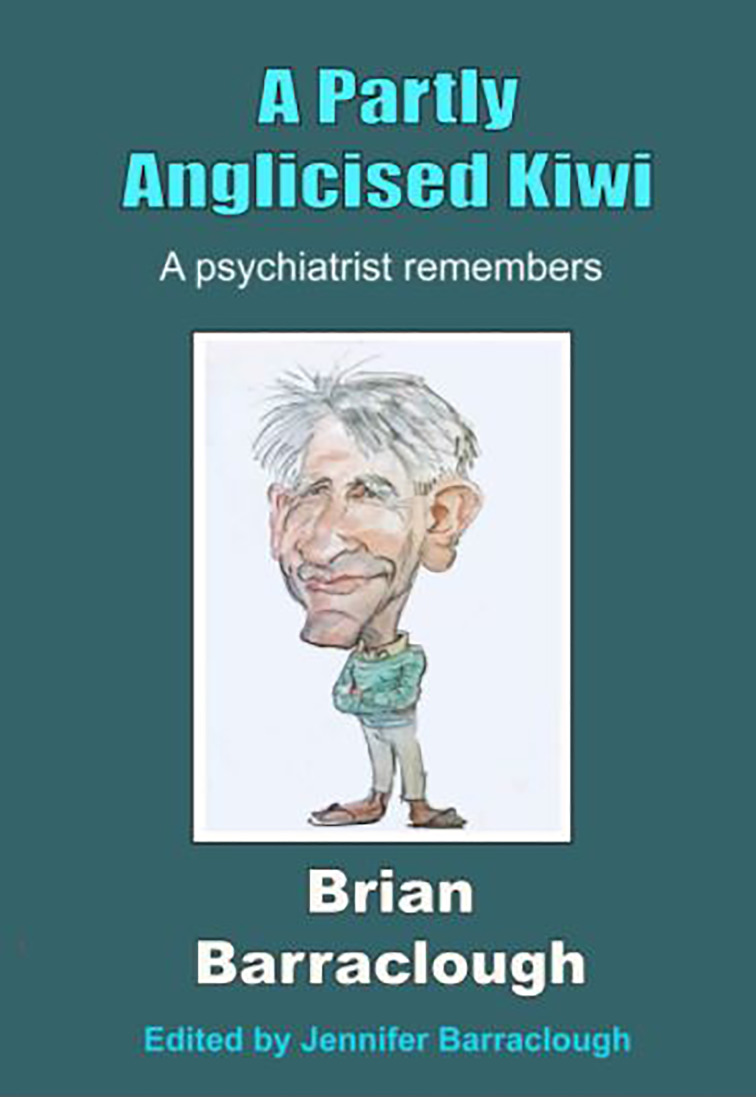

Psychiatrists are now interesting to the general public. It was not always so. After years of meeting them only as cardboard cut-out figures in crime novels there is now curiosity about who they are and what they do. I have just finished reading Joanna Cannon's book *Breaking and Mending*, about the effect of psychiatry on the person. Written by someone exquisitely sensitive to every nuance in professional life, Joanna's book was an eye-opener for me with my more hardened exterior. Brian Barraclough's autobiography is very different. It satisfies the voyeur in the reader – what are the people psychiatrists treat really like and what do they get up to?

And there is much for the voyeur here. But this independently published book has a misleading title. Brian Barraclough does not come over as ‘partly Anglicised’; he is a Kiwi through and through, and although his assiduous English wife Jennifer, also a well-known psychiatrist, has done some, possibly a great deal of, editing she cannot hide its craggy authenticity. Despite spending 38 years of his life in the soft underbelly of Sussex and Hampshire, Dr Barraclough has not acquired the veneer of many English sophisticates, who might write more smoothly. He has always lived in the marvellous upside down map of the world where New Zealand is securely on the top.

Here we read about the recent history of psychiatry. It is so odd that it is hard to believe it is recent and not ancient history. Barraclough fosters the old by giving an oral testimony, almost mediaeval in its bluntness, unapologetic and unadorned, thinking primarily not of the reader but of the need for accurate report. So, we are taken back to characters and experiences that some younger psychiatrists might find unbelievable. Dr Barraclough's mentor, Harold Bourne, bluntly tells a female medical student ‘your vivid red lipstick represents a penis’, and also, while maintaining he is primarily a psychoanalyst, practises long-term ‘ECT maintenance’ (i.e. giving courses of electroconvulsive therapy at intervals of a few weeks ‘to avoid relapse’). We read about a woman who broke her teeth after unmodified ECT (she had osteogenesis imperfecta), another woman, a devout member of the Church of England who had severe obsessional disorder, who, after much deliberation, was recommended for a leucotomy. Afterwards she is observed by Barraclough to squat and defecate on the floor. She looks up and says, ‘That is my shit, clean it up’. He also describes working for a psychiatrist who carried out ‘behaviour change treatment’ for homosexual men in a closed ward for 9 days, topped up with aversion therapy for others; there is no discussion of outcome.

You might think that these experiences might all come from New Zealand. No, half come from the Mecca of psychiatry in the 1960s, the Maudsley Hospital in London. This was the ‘only decent place to study’, according to Harold Bourne, where once trained, the inspired joined the diaspora to spread enlightenment to the hungry elsewhere. But this was a time when opinion, not evidence, guided clinical practice, even at the Maudsley. After all these experiences, it is not surprising that Dr Barraclough turned his attention to the dead, to the subject of suicide, where his contribution is widely praised and may be described more fully in a forthcoming volume.

